# Multimodal imaging in constrictive pericarditis: a case report of cardiac cirrhosis

**DOI:** 10.47487/apcyccv.v4i3.294

**Published:** 2023-09-30

**Authors:** Lucía Barriales-Revilla, Lindsay Benites-Yshpilco, Roberto Baltodano-Arellano, Luis Falcón-Quispe, Kelly Cupe-Chacalcaje, Angela Cachicatari-Beltrán, Gerald Lévano-Pachas

**Affiliations:** 1 Servicio de Cardiología, Hospital Guillermo Almenara Irigoyen, EsSalud, Lima, Peru. Servicio de Cardiología Hospital Guillermo Almenara Irigoyen, EsSalud Lima Peru; 2 Facultad de Medicina, Universidad Nacional Mayor de San Marcos, Lima, Peru. Universidad Nacional Mayor de San Marcos Facultad de Medicina Universidad Nacional Mayor de San Marcos Lima Peru; 3 Unidad de Imágenes Cardiacas, Servicio de Cardiología, Hospital Guillermo Almenara Irigoyen, EsSalud, Lima, Peru Unidad de Imágenes Cardiacas, Servicio de Cardiología Hospital Guillermo Almenara Irigoyen, EsSalud Lima Peru

**Keywords:** Constrictive pericarditis, Pericardiectomy, Echocardiograph, Cirrhosis, Pericarditis Constrictiva, Cirrosis Cardiaca, Pericardiectomía, Ecocardiografía

## Abstract

La pericarditis constrictiva es una causa infrecuente de ascitis y cirrosis cardiaca. Presentamos el caso de un paciente varón de 36 años, con antecedentes de cirrosis de etiología desconocida, que consultó por ascitis refractaria, disnea y edema de miembros inferiores. La ecocardiografía determinó pericarditis constrictiva, que fue corroborada por los hallazgos de la tomografía computarizada. El empeoramiento clínico y hemodinámico del paciente condujo a una pericardiectomía urgente con recuperación satisfactoria. Este informe muestra una consecuencia clínica grave de la pericarditis constrictiva, la cirrosis cardiaca, que fue reversible con la extirpación del pericardio. La imagen multimodal fue esencial en el diagnóstico de la pericarditis constrictiva.

## Abstract (en)

Constrictive pericarditis is a rare cause of ascites and cardiac cirrhosis. We present the case of a 36-year-old male patient with a history of cirrhosis of unknown etiology, who consulted for refractory ascites, dyspnea, and lower limb swelling. Echocardiography determined constrictive pericarditis, which was corroborated by the findings of computed tomography. The clinical and hemodynamic worsening of the patient led to an emergency pericardiectomy with satisfactory recovery. This report shows a severe clinical consequence of constrictive pericarditis, cardiac cirrhosis, which was reversible with pericardial extirpation. Multimodal imaging was essential in the diagnosis of constrictive pericarditis.

## Introduction

Constrictive pericarditis is an infrequent cause of right heart failure, characterized by rigid, thick, fibrotic, and/or calcified pericardium. In advanced stages, it leads to congestive hepatomegaly and refractory ascites ^1^. Early diagnosis is often challenging due to the absence of specific symptoms and signs of this entity and its insidious course. The delay in diagnosis can even lead to liver transplantation in inadvertent cardiac cirrhosis ^2^. Imaging studies currently determine the non-invasive diagnosis of this entity. The two objectives of this document are to highlight constrictive pericarditis as a potential cause of cirrhosis and mainly to show the importance of multimodal imaging for the detection of constrictive pericarditis.

## Case report

A 36-year-old male patient attended the emergency room due to refractory ascites, recently associated with dyspnea and swelling of the lower limbs. Medical history highlighted hepatic cirrhosis with multiple admissions for ascites. The most relevant findings on physical examination were severe jugular ingurgitation, ascites, and a positive fovea sign on both legs. The laboratory tests stood out for the alteration in liver tests with hypoalbuminemia (2.2 g/dl) and elevation of enzymes: alkaline phosphatase (1319 IU/l) and gamma-glutamyl transpeptidase (1328 IU/l).

The liver Doppler ultrasound reported hepatosplenomegaly, signs of diffuse chronic liver disease, dilation of suprahepatic veins, and inferior vena cava, while flowmetry was suggestive of post-hepatic portal hypertension of probable cardiac etiology.

**Figure 1:**
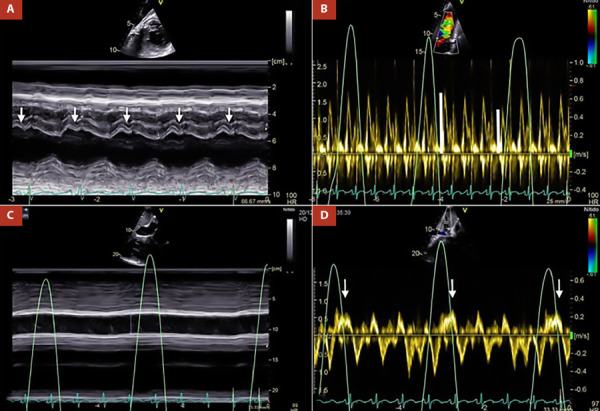
A. M mode of interventricular septum. Pericardial notch is evident (white arrows), B. Pulsed Doppler at the level of the tricuspid valve. There is transtricuspid variability of 25% (white lines), C. M mode of the inferior vena cava, which is markedly dilated without respiratory variability, D. Pulsed Doppler of the suprahepatic vein, reverse diastolic flow is shown in expiration (white arrows).

Transthoracic echocardiography (TTE) showed suboptimal images due to the poor acoustic window; however, some relevant variables were determined. The pericardial notch in the M mode of the interventricular septum **(**Figure 1A**)** and the respiratory variability in the trans tricuspid flow were evidenced **(**Figure 1B**)**. Likewise, in the subcostal approach, pericardial effusion was ruled out, however, the dilation of the inferior vena cava without respiratory collapse **(**Figure 1C**)**, and finally reverse diastolic flow on expiration in the suprahepatic vein determined a constrictive physiology **(**Figure 1D**)**.

**Figure 2:**
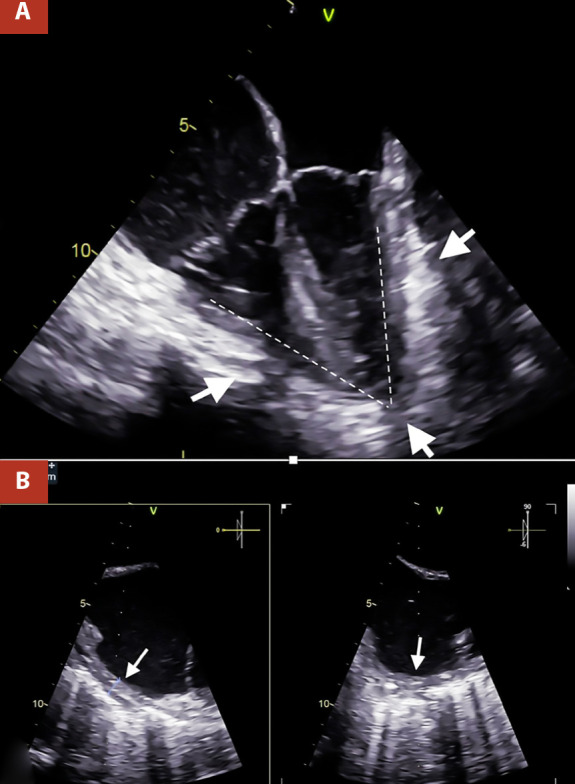
A. Two-dimensional TEE 4 cameras. Thickened pericardium (white arrows) is visualized, which markedly limits ventricular compliance, causing the ventricles to present an arrowhead morphology, B. Orthogonal TEE images, focused on the lateral wall of the right atrium with calcification evident by posterior acoustic shadowing, the pericardial thickness was 7 mm (white arrows).

The most important evidence in the transesophageal echocardiography (TEE), was the inability of both ventricles to achieve complete distensibility due to the surrounding pericardial shell, which led to an arrowhead morphology of the heart **(**Figure 2A and Video 1). The pericardial thickness was measured at 7 mm in the right atrial wall, which also presented a notorious limitation in its distensibility (Figure 2B  and Video 2).

**Figure 3:**
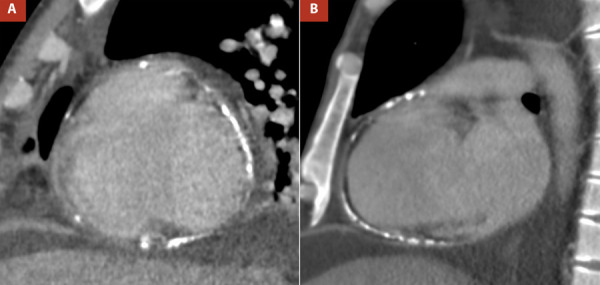
A,B Thoracic radiography, anteroposterior view, showed pulmonary congestion and right pleural effusion, C. Non-contrast non-gated computed tomography reformat revealed thickened pericardium (3.7 mm) with diffuse calcification, D. Computed tomography sagittal reformat demonstrated calcified pericardium involving right chambers.

In the chest images, the radiograph in the context of pulmonary congestion did not show calcification in the pericardium **(**Figure 3A-B**)** however at another time, the non-contrast computed tomography (CT) confirmed diffuse thickening of the pericardium, with evidence of calcification predominantly in the right cavities **(**Figure 3C-D**)** and ruled out parenchymal lung problems.

**Figure 4:**
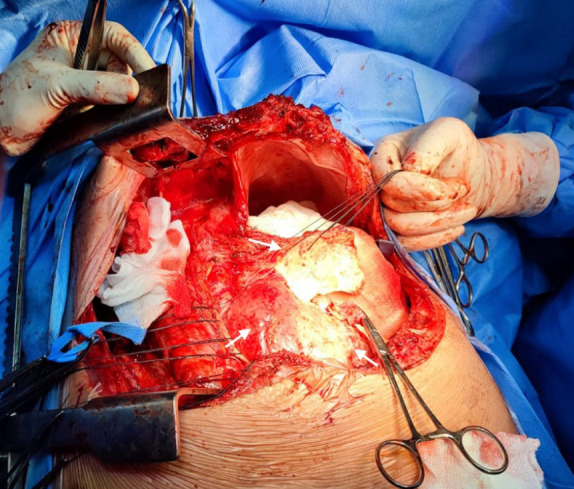
Clamshell approach. Exposure of the pericardium, which appears severely thickened and calcified (white arrows), which correlates with TEE.

The torpid clinical evolution conditioned the emergency surgical intervention of total pericardiectomy **(**Figure 4**)**. A Clamshell incision was performed without extracorporeal circulation, finding empyema, hemothorax, severely adherent pulmonary cortex and thickened and calcified parietal pericardium with lax epicardial-pericardial adhesions around the heart and great vessels. No complications were reported. The postoperative period was satisfactory with early extubation and successful recovery with early discharge. The histology concluded fibrosis, vascular congestion, and chronic inflammatory infiltrate. On immunohistochemistry, the Ziehl-Neelsen stain for tuberculosis was negative.

At three months follow-up, the patient was asymptomatic. Control of the liver profile showed normalization of albumin (4.6 g/dl), while liver enzymes decreased substantially (alkaline phosphatase, 759 IU/l and gamma-glutamyl transpeptidase (572 IU/l).

## Discussion

Constrictive pericarditis, characterized by an increase and equalization of the end-diastolic pressures of the four chambers ^3^, is a stealthy and rare entity with critical clinical and hemodynamic consequences, from right heart failure to cardiac cirrhosis ^4^, as occurred in our patient. In extreme cases, patients can develop cachexia and loss of muscle mass ^3^. The etiology is diverse, including viral causes, thoracic surgeries, collagen diseases, and radiotherapy ^5^, and in low-income countries tuberculosis is an important cause to consider ^6^. This case was compatible with an idiopathic origin.

The diagnosis of this entity is challenging. The clinical presentation of this case was basically dominated by refractory ascites and alterations in the liver profile, which pointed towards a primarily liver problem. Unfortunately, jugular ingurgitation, a sign previously reported as more sensitive in constrictive pericarditis (up to 83% of cases) ^7^, was unnoticed initially in the patient’s clinical examination.

The TTE is the first imaging test and is usually diagnostic in most cases. The use of the M mode shows interventricular septum movement with the respiratory cycle, which represents pathological ventricular interdependence ^5^. However, the most specific sign of constrictive pericarditis consists of a sudden anterior displacement of the interventricular septum followed by a sudden posterior rebound (septal shudder or pericardial notch), also demonstrated in M mode ^8^^,^^9^. In two-dimensional mode, a variable that represents elevated right atrial filling pressures is dilatation of the inferior vena cava without respiratory collapse ^5^. On the other hand, the Doppler study demonstrates restricted biventricular filling with a rapid deceleration of early filling velocity (E wave) and a small or absent A wave. Likewise, there is a substantial respiratory variability in mitral (>25%) and tricuspid (>40%) filling speed ^5^. In the study of the suprahepatic veins, diastolic reflux is typically evidenced in expiration, the more specific Doppler variable for constrictive pericarditis ^10^^,^^11^. The presence of all these variables in the patient’s echocardiography determined the diagnosis of constrictive pericarditis. Finally, it is important to mention that the marked respiratory variability and the normal early diastolic mitral annulus velocity (e) (>8 cm/s) distinguish it from restrictive myocardiopathy ^5^.

The main utility of the TEE in this entity is to measure the pericardial thickness with greater sensitivity and accuracy than the transthoracic approach ^12^. However, this procedure was primarily indicated by poor transthoracic acoustic window in our patient. The TEE in addition to demonstrating increased pericardial thickness, evidenced the limited distensibility of the four chambers, verifying the severity of this entity. This study’s additional relevant and unprecedented finding is the arrowhead morphology of both ventricles together, due to the pericardial shell.

The CT is not usually as the first imaging modality for constrictive pericarditis; however, in some settings, such as end-stage calcified pericardial constrictions, it is essential to determine the location and extent of pericardial calcification ^13^, which was done in this case. In addition, chest tomography provides an assessment of concomitant lung disease and the proximity of cardiovascular structures to the sternum. It should be mentioned that the absence of pericardial thickening by tomography does not exclude the diagnosis of constrictive pericarditis ^14^. Among cardiac imaging studies, cardiovascular magnetic resonance imaging (CMR) is very sensitive for the diagnosis of constrictive pericarditis, visualizing anatomical structures such as pericardial thickening on T1, in addition to the presence of pericardial edema on T2. Otherwise, CMR can provide useful information on the flattening of the septum with respiration through cine images in real-time ^15^^,^^16^. Therefore, CMR may be useful in the preoperative evaluation of constrictive pericarditis. In our case, this study was not necessary because the patient already had a definitive diagnosis.

In conclusion, cardiac cirrhosis due to constrictive pericarditis is potentially reversible with pericardium removal. Multimodal imaging confirmed the diagnosis in this case; however, the TTE was crucial in the initial approach.
